# Supplementary material for: HIVprotI: an integrated web based platform for prediction and design of HIV proteins inhibitors
Source: J Cheminform. 2018 Mar 9;10:12. doi: 10.1186/s13321-018-0266-y (PMC5845081; doi:10.1186/s13321-018-0266-y)
Supplement: Supplementary file 2 — Additional file 2. Source code of HIVProtI web server. [file 13321_2018_266_MOESM2_ESM.zip › HIVprotI_Source-code/hivprotI/jsmol/images.htm]

JSmol -- Jmol/HTML5 Demo


|  |  |
| --- | --- |
|  |  |
|  | |

This page demonstrates some relatively new capabilities
in Jmol relating to incommensurately modulated structures
(msCIF data). After clicking on atoms, you will get a
FLOT graph that you can click on to set a specific t-value.
Some interesting Jmol features used here include
easy manipulation of data arrays for tabulation, the animated
GIFS created using the CAPTURE command, dynamic
measurements, and a few more goodies that you can find
in plott.spt." -- Bob Hanson Dec 12, 2013/May 4, 2014
